# Mutualism–parasitism paradigm synthesized from results of root-endophyte models

**DOI:** 10.3389/fmicb.2014.00776

**Published:** 2015-01-12

**Authors:** Keerthi G. Mandyam, Ari Jumpponen

**Affiliations:** ^1^Department of Agriculture, Alcorn State UniversityLorman, MS, USA; ^2^Division of Biology, Ecological Genomics Institute, Kansas State UniversityManhattan, KS, USA

**Keywords:** *Arabidopsis thaliana*, dark septate endophyte, mutualism, parasitism, population inference, symbiosis

## Abstract

Plant tissues host a variety of fungi. One important group is the dark septate endophytes (DSEs) that colonize plant roots and form characteristic intracellular structures – melanized hyphae and microsclerotia. The DSE associations are common and frequently observed in various biomes and plant taxa. Reviews suggest that the proportion of plant species colonized by DSE equal that colonized by AM and microscopic studies show that the proportion of the root system colonized by fungi DSE can equal, or even exceed, the colonization by AM fungi. Despite the high frequency and suspected ecological importance, the effects of DSE colonization on plant growth and performance have remained unclear. Here, we draw from over a decade of experimentation with the obscure DSE symbiosis and synthesize across large bodies of published and unpublished data from *Arabidopsis thaliana* and *Allium porrum* model systems as well as from experiments that use native plants to better resolve the host responses to DSE colonization. The data indicate similar distribution of host responses in model and native plant studies, validating the use of model plants for tractable dissection of DSE symbioses. The available data also permit empirical testing of the environmental modulation of host responses to DSE colonization and refining the “*mutualism-parasitism-continuum*” paradigm for DSE symbioses. These data highlight the context dependency of the DSE symbioses: not only plant species but also ecotypes vary in their responses to populations of conspecific DSE fungi – environmental conditions further shift the host responses similar to those predicted based on the *mutualism-parasitism-continuum* paradigm. The model systems provide several established avenues of inquiry that permit more detailed molecular and functional dissection of fungal endophyte symbioses, identifying thus likely mechanisms that may underlie the observed host responses to endophyte colonization.

## INTRODUCTION

Dark septate endophyte (DSE) fungi colonize plant roots and form characteristic structures – melanized hyphae and microsclerotia – and often have variable effects on plant growth. This inter- and intraspecific variability in host responses has been hypothesized to be central to plant community structuring by mycorrhizal fungi (Wilson and Hartnett, 1998; Hartnett and Wilson, 1999; van der Heijden, 2002). Similarly, the variability in host responses to DSE fungi may promote selection mosaics proposed for ectomycorrhizal symbioses (Piculell et al., 2008).

An issue that has remained under continuous debate is whether the DSE symbiosis should be considered beneficial to the host plant or rather as a weak parasitism (Jumpponen, 2001; Addy et al., 2005; Mandyam and Jumpponen, 2005; Alberton et al., 2010; Newsham, 2011; Mayerhofer et al., 2013). The general host responses to DSE fungi have remained difficult to discern, partly because of their wide variability, partly because of independent small studies that draw conclusions based on a limited number of fungal individuals. Here we aim to synthesize various bodies of data to better resolve the host responses to the colonization by these abundant fungi as well as to discern some abiotic controls that may lead to shifts in these observed host responses. Results from studies that use model and native plant systems provide unique empirical insights into the variability in host responses to DSE fungi drawn from populations of conspecific fungi. We argue that these data permit empirical evaluation of the “*mutualism-parasitism-continuum*” paradigm (Johnson et al., 1997; Saikkonen et al., 1998). We conclude by describing a general neutral null-hypothesis of host responses to fungal symbionts applicable beyond the DSE symbiosis. The mutualism–parasitism paradigm has been used as a general framework to understand the mycorrhizal symbioses that have – similarly to DSE symbioses – been considered variable when observed in different hosts or compared under different abiotic conditions.

## DARK SEPTATE ENDOPHYTES – WHAT ARE THEY?

Research on DSE fungal has a long history. Melin (1922) described a melanized sterile fungus – *Mycelium radicis-atrovirens* – that he isolated from ectomycorrhizal roots of conifers. These isolates colonized roots intracellularly, suggesting an association distinct from ectomycorrhizae. To emphasize the distinction from mycorrhizas, Melin called this association a “*pseudomycorrhiza.*” More recently, similar melanized root-associations have been reported from a vast variety of host plants (>600 plant species representing >100 families), biomes, and ecosystems (Jumpponen and Trappe, 1998b; Mandyam and Jumpponen, 2005; Kageyama et al., 2008). The lists of plants with such root-colonization have been expanded with each study that records the presence of indicative structures within host roots (e.g., Kovacs and Szigetvari, 2002).

Dark septate endophytes are a miscellaneous group of mainly ascomyceteous root-colonizing fungi characterized by melanized cell walls and intracellular colonization of healthy plants (Jumpponen and Trappe, 1998b). Early stages of intracellular colonization often include non-pigmented hyphae into which the melanins are deposited later. These difficult to visualize hyphae (see Barrow and Aaltonen, 2001; Barrow, 2003) may also indicate different consortia of root-inhabiting fungi altogether (Porras-Alfaro et al., 2008; Khidir et al., 2010). In addition to potentially biome specific fungal guilds and inconsistent semantics, the research on root-associated endophytes is further burdened by lack of taxonomic cohesion, polyphyletic evolutionary origins of the DSE fungi, and their variable ecological or physiological functions (Caldwell et al., 2000; Addy et al., 2005; Grünig et al., 2008). However, the DSE fungi form melanized inter- and intracellular hyphae and melanized microsclerotia that are indicative and characteristic morphological structures in the host roots (Jumpponen and Trappe, 1998b; Rodriguez et al., 2009; Mandyam et al., 2010).

## ABUNDANCE OF DSE FUNGI

Compared to better known mycorrhizal symbioses or the vertically transmitted systemic foliar endophytes, the root-associated fungal endophytes have received very little attention (Rodriguez et al., 2009). This is a serious gap in our understanding of the fungal associations, because the DSE fungi are common in many ecosystems including those in the Antarctic, Arctic, boreal, subtropical, and temperate regions (Mandyam and Jumpponen, 2005; Kageyama et al., 2008). The research gap is further highlighted by studies that compare host colonization by the root endophytes and mycorrhizal fungi in various habitats. The rare studies that estimate the root colonization by both mycorrhizal and endophytic fungi indicate that the DSE fungi are possibly as abundant as mycorrhizas (Mandyam and Jumpponen, 2008; Dolinar and Gaberscik, 2010; Zhang et al., 2010), if not more so (Mandyam and Jumpponen, 2008). Despite their apparent great abundance, functions of the DSE fungi, particularly their general effects on the colonized hosts, have not been resolved.

## *MUTUALISM-PARASITISM-CONTINUUM* PARADIGM

The mechanisms and their magnitudes that alter interspecific interactions are central in ecology (Thompson et al., 2001). Research on mycorrhizal fungi has been pivotal in developing an understanding of the variability in presumed mutualisms (Sapp, 2004). The “*mutualism-parasitism-continuum*” is a paradigm established as a framework to explain why symbiotic associations may deviate from mutualisms to parasitisms (Francis and Read, 1995; Saikkonen et al., 1998; Jones and Smith, 2004). According to this paradigm, compatible host-fungus associations produce host responses that are flanked at one end by obligate mutualisms in which hosts fail to survive in absence of their fungal partners and at another end by parasitisms that lead to the death of a host plant. While the position of each compatible host-fungus association along this continuum is interesting and perhaps context-dependent (Karst et al., 2009), it is imperative that we understand the underlying controls of the variability in these symbioses. These controls include, but are not limited to, biotic variability of the component fungi (Munkvold et al., 2004; Grünig et al., 2008; Mandyam et al., 2012, 2013) or host plants (Jones et al., 1990; Thomson et al., 1994; Karst et al., 2009; Hoeksema et al., 2010) as well as abiotic variability in the availability of light or nutrients or in the stress under which the host-fungus symbiosis is evaluated (Johnson et al., 1997; Redman et al., 2001; Rodriguez et al., 2008; Johnson, 2010).

We describe, reanalyze, and synthesize studies conducted utilizing model plant systems and then use those data to infer general host responses to DSE fungi. We further evaluate the applicability of these model plant systems via comparisons with native plants. Our data clearly indicate that while the host species identities are important, so are the host and fungal genotypes and broad functional groupings (e.g., forb vs. grass; Mandyam et al., 2012). Additional experiments indicate – consistently with predictions of the mutualism-parasitism-continuum framework – that host responses in these associations can be modulated by abiotic conditions.

## HOST RESPONSES TO DSE

The DSE fungi may either inhibit or enhance host plant growth (Jumpponen, 2001; Mandyam and Jumpponen, 2005; Grünig et al., 2008; Alberton et al., 2010; Newsham, 2011; Mandyam et al., 2012, 2013; Mayerhofer et al., 2013). The mechanisms that lead to the variable host responses are uncertain but often speculated in conjunction with inoculation experiments. Similarly to both arbuscular mycorrhizal and ectomycorrhizal symbioses where host responses have been considered context-dependent (Karst et al., 2008; Hoeksema et al., 2010), host responses to DSE fungi vary between host species and between coarse functional groupings (Mandyam et al., 2012). In contrast to interspecific variability, intraspecific variability is often discussed but rarely addressed (Piculell et al., 2008; Karst et al., 2009; Mandyam et al., 2013). Empirical evaluation of host responses within and among species to populations of conspecific fungi allow for assessment of intraspecific components of both hosts and fungi in DSE symbiosis (Mandyam et al., 2013).

In addition to the inter- and intraspecific variability among the plants (Piculell et al., 2008; Karst et al., 2009) and fungal symbionts (Munkvold et al., 2004; Mandyam et al., 2012), the potential drivers of the variable host responses – whether negative or positive – include competition with more serious root parasites and pathogens, facilitation of host nutrient uptake, or modulation by environmental stressors such as shade, drought, salinity, and nutrient depletion (Johnson et al., 1997; Kageyama et al., 2008; Rodriguez et al., 2008; Hoeksema et al., 2010). Endophyte competition with antagonistic fungi is evidenced by the upregulation of plant defense pathways as a result of endophyte colonization (Mandyam and Jumpponen, 2014) and may lead to growth promotion if the cost of combined colonization is lesser than the cost of antagonist colonization alone (Mandyam and Jumpponen, 2005). Like in mycorrhizal symbioses (Hoeksema et al., 2010), facilitation of nutrient uptake is supported by increases in N or P contents and concentrations in the tissues of inoculated hosts (Jumpponen et al., 1998; Newsham, 2011). While this function is attractive mechanism for the growth stimulation in DSE symbiosis (see Newsham, 2011), it suffers from lack of evidence for any perifungal interface through which the nutrient exchange between the host and fungus would take place (Yu et al., 2001). Finally, analogously to the symbiosis between *Curvularia* and *Dichanthelium* (Redman et al., 1999), other endophytes – including DSE – may lead to modulation of plant environmental tolerances (Mandyam and Jumpponen, 2005) that may improve survival and performance during periods of stress.

Generalizations about the functional attributes of DSE fungi are complicated by their taxonomic diversity and the limited overlap in the communities across biomes (Addy et al., 2005; Kageyama et al., 2008; Herrera et al., 2010a). Recent meta-analyses of a limited number of available studies (Alberton et al., 2010; Mayerhofer et al., 2013) suggested that while host growth responses to colonization by DSE fungi were variable, they tended to be negative. In contrast to those meta-analyses, Newsham (2011) concluded that the outcomes of the DSE inoculation depended on the form of nitrogen supplied during the experiment (organic vs. inorganic) highlighting again the environmental context dependency of the symbiosis. Similarly to these conclusions, Mayerhofer et al. (2013) underline the impact of experimental designs or conditions that may confound the observed variability in plant responses. Overall, the three meta-analyses on the functional attributes of the DSE fungi indicate the difficulty of providing strong and meaningful conclusions on the DSE symbiosis highlighting the importance of ambitious empirical studies that evaluate broader selections of hosts and fungi under consistent experimental conditions. The difficulty of arriving at meaningful conclusions is further exaggerated by the diversity of distinct unrelated fungi involved in these associations. Furthermore, predictions on the relative importance of different environmental parameters that may modulate the host responses stem from isolated studies that use small subsets of plants and often only one or two strains of fungi. We urge the use of large numbers of conspecific fungal strains in more ambitious tractable empirical studies that use model plants followed by confirmatory experiments that utilize native plants.

## DEBATE ON HOST RESPONSES TO DSE COLONIZATION

Because of the contrasting results from experiments in which host plants are inoculated with the DSE fungi, their effect on the host performance has remained open to debate. Jumpponen (2001) proposed that because these associations lead to host responses ranging from inhibition of growth and performance to occasionally substantial increases in growth, the DSE symbiosis should be considered similarly to mycorrhizal associations. This argument relies on the “*mutualism-parasitism-continuum*” paradigm. Addy et al. (2005) reviewed the fungal associations best exemplifying the DSE symbioses and concluded that – in contrast to Jumpponen (2001) – the DSE fungi are more appropriately characterized as weak parasites than as mutualists within the *mutualism-parasitism-continuum*. The absence of host-derived perifungal membrane and its interfacial matrix structurally support this argument. While meta-analyses (Alberton et al., 2010; Mayerhofer et al., 2013) that summarized results from inoculation experiments concluded that on average the DSE tended to reduce host growth, others (Newsham, 2011) have provided contrasting conclusions. Perhaps the underlying reasons for these contrasts lies indeed in the variability in the experimentation (Mayerhofer et al., 2013).

Here, we contribute to this debate by drawing from more than a decade of continuous research effort and synthesize large bodies of accumulated published and unpublished data. We include a number of concerted, uniform experiments utilizing *Allium* and* Arabidopsis* models; complementary experiments with native hosts; and, experiments that evaluate the environmental modulation of the symbiosis. While this synthesis focuses explicitly on the DSE symbiosis, the neutral null hypotheses, the population-centered approaches, and the environmental modulation of the symbioses are broadly applicable to other symbiotic systems.

## MODEL PLANT RESPONSES TO INOCULATION WITH DSE FUNGI

We define the DSE symbiosis narrowly and consider only those species or fungal strains that form the characteristic DSE structures (i.e., intracellular microsclerotia). This approach omits many hyaline root-associated fungi (RAF) that have been frequently observed, particularly in (semi-)arid ecosystems (e.g., Herrera et al., 2010b). As a result, we primarily focus on *Periconia macrospinosa* and its close relatives from the prairie ecosystems (Mandyam et al., 2010) and acknowledge that our experiments do not include other common DSE fungi such as *Phialocephala fortinii* or *Cadophora finlandica* (formerly *Phialophora finlandica*) that tend to be common in boreal/temperate forest ecosystems (Jumpponen and Trappe, 1998b; Jumpponen, 2001; Grünig et al., 2008).

All fungi isolated from host roots neither produce DSE structures nor stimulate host growth (Jumpponen, 2001; Kageyama et al., 2008; Mandyam et al., 2010; Knapp et al., 2012). While some of the fungi isolated from roots behave like pathogens (Kageyama et al., 2008; Mandyam et al., 2010; Tellenbach et al., 2011), the commonly isolated DSE species tend to lead to host responses that range from growth inhibition to growth stimulation as one would predict based on the *mutualism-parasitism-continuum* paradigm. It is the heterogeneity of the fungi that can be isolated from the roots or detected in them molecularly that presents a challenge in the endophyte research. Isolation of fungal strains and fulfilling the Koch’s postulates are mandatory steps to convincingly confirm that acquired isolates are indeed responsible for producing the indicative DSE structures in the roots (Mandyam et al., 2010; Jumpponen et al., 2011a; Knapp et al., 2012). Molecular studies of the root-associated fungal communities particularly suffer from the inability to unequivocally detect endophytes (Jumpponen et al., 2011a,b), despite their occasionally high occurrence in many plant species (Mandyam and Jumpponen, 2008; Mandyam et al., 2012).

A typical experiment in which hosts have been inoculated with DSE fungi includes only very few fungal strains (e.g., Jumpponen and Trappe, 1998a; Jumpponen et al., 1998; Newsham, 1999; Vohnik et al., 2005; Usuki and Narisawa, 2007; Hou and Guo, 2009; Yuan et al., 2010) and only recently have more ambitious studies that include multiple hosts and/or fungal strains emerged (see Mandyam et al., 2012, 2013; Tellenbach and Sieber, 2013). Comparisons of conspecific individuals within common DSE species (*Periconia*) from tallgrass prairie clearly indicate that inoculation with different fungal individuals leads to different host responses in model (Mandyam et al., 2013) and non-model systems (Mandyam et al., 2012). Similarly, host responses to Helotialean DSE fungi also differ supporting the notion that there are differences among the DSE species (Jumpponen and Trappe, 1998a; Jumpponen, 2001; Tellenbach et al., 2011). While inoculation experiments may suffer from limited inferential capacity and extrapolation to natural conditions, these controlled experiments are mandatory to better understand host responses in absence of complex biotic and abiotic interactions.

**FIGURE 1:**
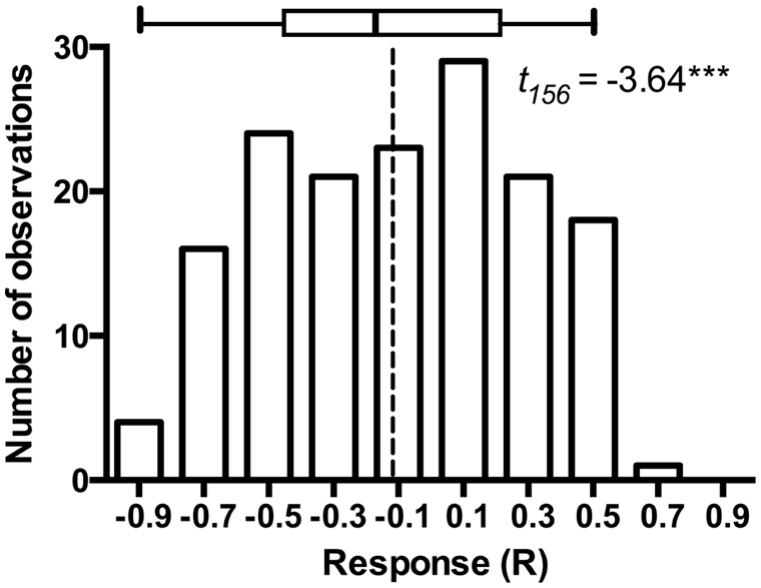
**Frequency distribution of *Arabidopsis* responses to inoculation in 157 experiments that paired *Arabidopsis thaliana* either inoculated with *Periconia macrospinosa* or with sterile fungal medium (mock-control).** The experimental procedures are described in full detail in Mandyam et al. (2013). Response (R) to inoculation indicates the difference between the control and inoculated plants relative to control (inoculated < control) or inoculated plants (control < inoculated; Klironomos, 2003). *t*-test on the mean of 157 experiments indicates that average response to inoculation is negative (*P* < 0.0001) suggesting thus an overall parasitic association. The box identifies median, quartiles and 95% confidence intervals. Dashed line identifies the mean response across all 157 experiments.

The model plant *Arabidopsis thaliana* is subject to colonization by a variety of bacterial (Bulgarelli et al., 2012) and fungal endophytes (Garcia et al., 2013), including fungi that occupy root and rhizosphere (Mandyam et al., 2013; Mandyam and Jumpponen, 2014). As such, *A. thaliana* and its endophytes may provide a model for exploring endophyte associations in a well-defined system (Garcia et al., 2013). Mandyam et al. (2013) utilized a closed petri plate system that permitted 6–8 weeks incubation of *A. thaliana* with a minimal contamination risk. These experiments standardly used pairs of experimental treatments that were either mock-inoculated with a disk from fungal medium (fungus-free control) or inoculated with *P. macrospinosa*. While such experiments are tedious to set up and demand substantial growth room capacity, they benefit greatly from simple statistical inference on the host responses to the presence of the endophyte fungus. Furthermore, these experiments easily lend themselves for advanced classroom settings. We were fortunate to conduct a total of 157 such experiments (a total of 3,140 experimental units) with the assistance of more than thirty senior undergraduate students at Kansas State University. These experiments lend further support to conclusions in Mandyam et al. (2013): while the model plant responses to a population of endophytes may be variable and include several examples of symbioses that enhance host growth, on average the host responses are negative and the host growth is inhibited relative to the fungus-free controls (**Figure 1**).

While the variability in host responses to different DSE species is expected, the intraspecific variability in host responses to inoculation has received far less attention. Published data indicate that host responses are variable, often ranging from reduction in host growth to significant increases in the host biomass (Fernando and Currah, 1996; Mandyam et al., 2010, 2012). We have explored this topic extensively using two model systems (*Allium porrum* and *A. thaliana*; Kageyama et al., 2008; Mandyam et al., 2010, 2013): data show substantial intraspecific variability, even when the host genetic background is controlled (Mandyam et al., 2013). Taken together, these data lay a unique empirical foundation that clearly shows the dangers of making conclusions about a diverse guild of fungi without including a broad enough sampling of individuals drawn from a given population.

## HOST CONTROL OF RESPONSES TO DSE INOCULATION

**FIGURE 2:**
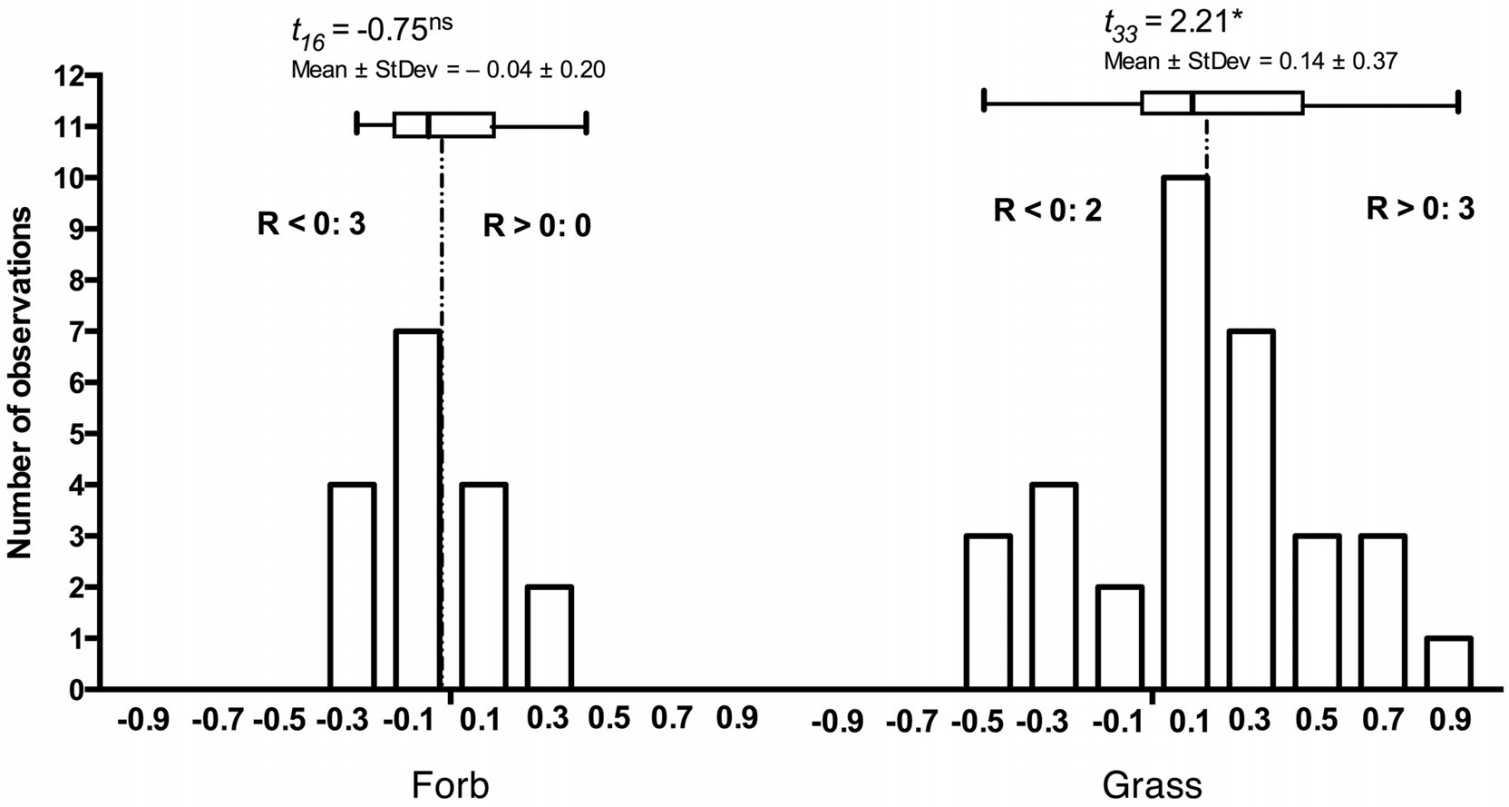
**Frequency distribution of responses in 17 forb and 33 grass *Periconia macrospinosa* inoculation experiments.** Each experiment paired experimental units inoculated either with *Periconia macrospinosa* or with sterile fungal medium (mock-control). The data were extracted from Mandyam et al. (2010, 2012) and analyses follow those described in Mandyam et al. (2013). Response (R) to inoculation indicates the difference between the control and inoculated plants relative to control (inoculated < control) or inoculated plants (control < inoculated; Klironomos, 2003). The grasses tended to respond positively (*t*-test, *P* < 0.05), whereas the forb response did not differ from zero. Inserts indicate the number of experiments where significant (ANOVA, *P* < 0.05) positive (R > 0) or negative (R < 0) responses were observed.

The principles that govern the assembly of host-specific endophyte communities from the general and more diverse soil communities remain poorly understood. Yet, co-occurring, adjacent hosts select root-associated community constituents from bulk soil so that the endophyte communities are distinct from bulk soil, lower in diversity (Lundberg et al., 2012; Bodenhausen et al., 2013), and may differ in composition among hosts. Naturally, host species differ in their susceptibility and responses to DSE (Mandyam et al., 2012). Although responses to root-associated endophytes often appear context-dependent, grass hosts are more extensively colonized in the laboratory and in the field when compared to dicotyledonous hosts (Mandyam et al., 2012). Similarly, the grass hosts tend to respond more positively to inoculation than forbs (**Figure 2**) suggesting that host responses may correlate with host evolutionary history or perhaps even suggest co-evolution of grasses and the abundant DSE fungi in grassland ecosystems.

**FIGURE 3:**
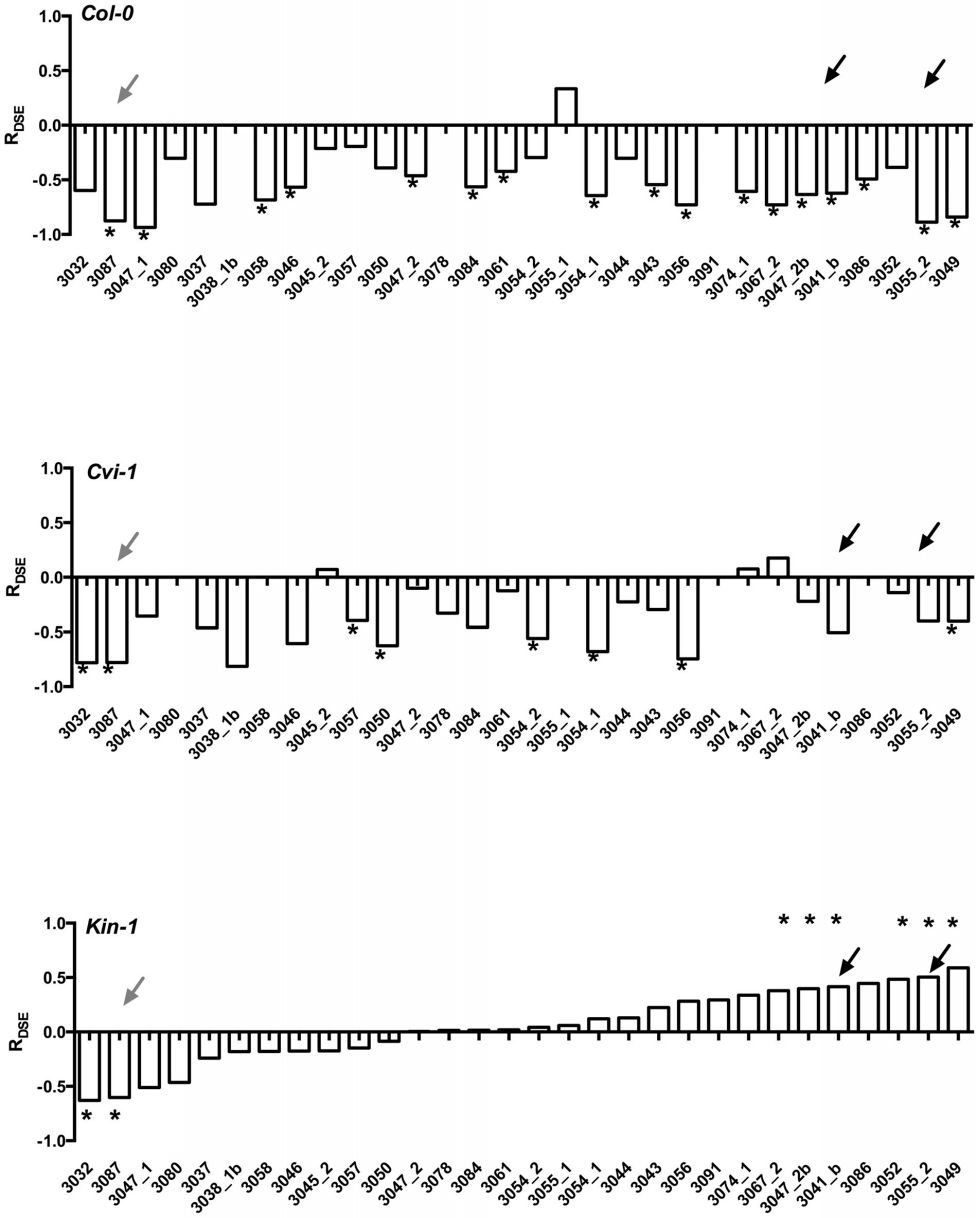
**Responses of three *Arabidopsis thaliana* accessions (*Col-0, Cvi-1, Kin-1*) to inoculation with 25 strains of *Periconia macrospinosa*.** The analyses follow those described in Mandyam et al. (2013). Response (R) to inoculation indicates the difference between the control and inoculated plants relative to control (inoculated < control) or inoculated plants (control < inoculated; Klironomos, 2003). Values above x-axis indicate a positive response, values below negative. Gray arrows indicate responses consistent across the three accessions, black arrows responses that range from negative to positive depending on the host accession. Asterisks indicate significant difference between the control and inoculated plants (ANOVA, *P* < 0.05). Figure is redrawn from Figure 2 in Mandyam et al. (2013).

Experiments with model plants indicate that not only do the host species differ in their responses, but also that *Arabidopsis* ecotypes that have very limited genotypic variability differ in their responses to DSE fungi (**Figure 3**). More importantly, it is rare that one fungal strain leads to similar host responses across different *Arabidopsis* accessions. Taken together, these findings suggest that host responses to DSE fungi vary among fungal strains and perhaps also among host genetic backgrounds. These findings clearly demonstrate that growth promoting fungal strains are present in environmental samples (Gentili and Jumpponen, 2006), but that the host responses may depend on the host genotype and are therefore often unpredictable.

## VALIDATION OF THE MODEL SYSTEM RESULTS WITH NATIVE PLANTS

**FIGURE 4:**
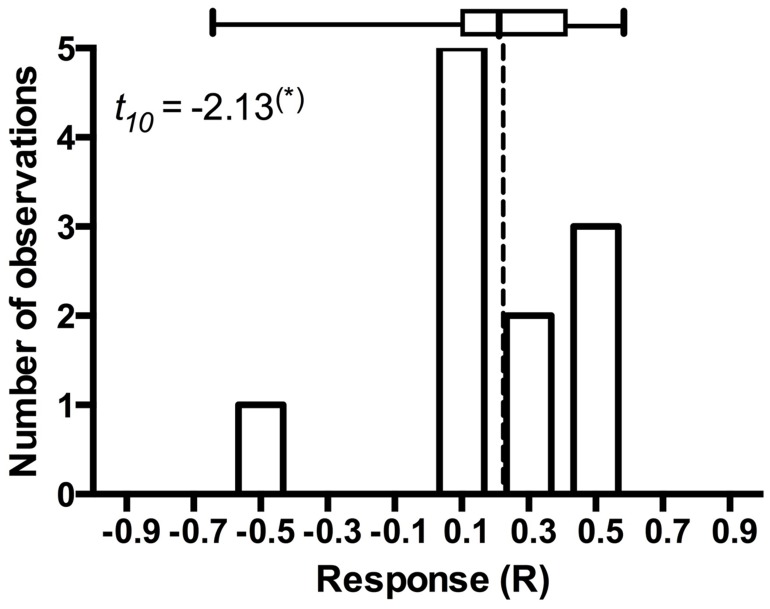
**Frequency distribution of *Andropogon gerardi* responses in eleven *Periconia macrospinosa* inoculation experiments.** The data were extracted from Mandyam et al. (2010, 2012). Response (R) to inoculation indicates the difference between the control and inoculated plants relative to control (inoculated < control) or inoculated plants (control < inoculated; Klironomos, 2003). Host response is marginally significantly (*t*-test, *P* < 0.10) positive and includes one potential outlier that deviates from the majority of experiments.

It is arguable whether the results from model plant systems apply to native hosts (Mandyam and Jumpponen, 2014). In addition to the experiments exploiting model plants (Mandyam et al., 2010, 2013; Mandyam and Jumpponen, 2014), we have conducted more limited experiments with eighteen native plant species common in the tallgrass prairie ecosystem where the fungal strains originate (Mandyam et al., 2010, 2012). While none of these datasets is quite as large as those accumulated with the *Allium* or *Arabidopsis* models, they nonetheless allow mapping of the native host responses into the *mutualism-parasitism-continuum* that serves as a central framework for this synthesis. These analyses demonstrate that the native plants span a range of responses similar to the model species (**Figure 2**), thus validating the predictions derived from the model systems. One of the native plants (the dominant native tallgrass prairie grass, *Andropogon gerardi*) allows analyses focusing on the responses to different conspecific strains of *P. macrospinosa* (**Figure 4**). These results indicate that – within a population of native conspecific host plants, host responses to DSE inoculation are as variable as they are in the model systems and span a full range from parasitism to mutualism. However, it is important to bear in mind that, across broader plant functional groupings, results indicate that none of the dicotyledonous hosts responded positively to inoculation with DSE fungi and three responded negatively (**Figure 2**). In contrast, three of the eight grass species responded positively, whereas two responded negatively (**Figure 2**). Taken together, our observations support the notion that grasses are more readily colonized by DSE fungi and that they tend to derive a greater benefit from the DSE symbioses than the forbs do.

## ENVIRONMENTAL MODULATION OF THE DSE SYMBIOSIS

**FIGURE 5:**
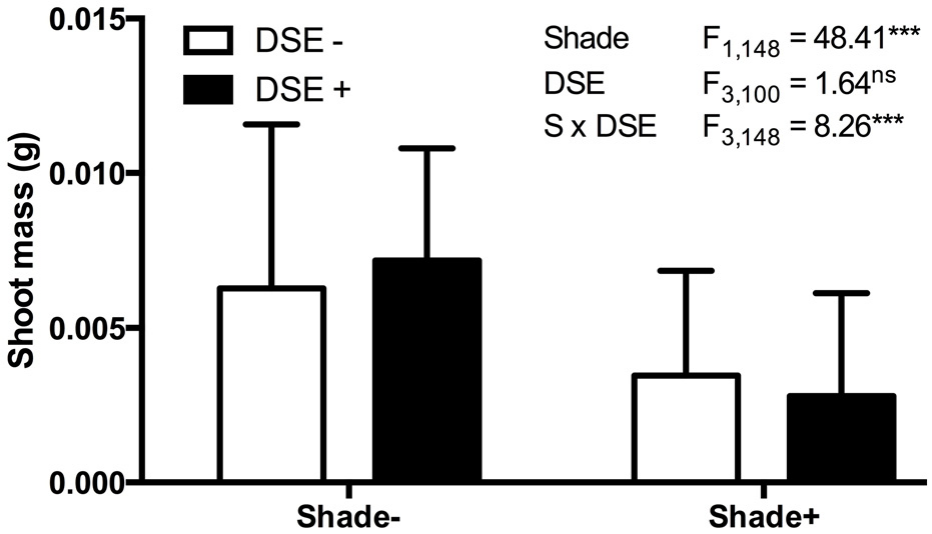
***Arabidopsis* biomass response to inoculation with *Periconia macrospinosa* and shading.** A total of 160 plants were included in paired experiments (*n* = 20 for each experiment), in which half were inoculated with living fungal culture, half with fungus free medium (see Mandyam et al., 2013 for details). Half of each inoculation treatment was covered with commercial shade cloth and half were left uncovered. Shoot biomass values were log_10_ transformed and analyzed for main effects (shade, inoculation) and their interaction in a mixed model ANOVA, where each paired experiment was assigned as a random effect. Significant shade and interaction terms (ANOVA, *P* < 0.0001) indicate lesser biomass accumulation in shade and suggest a greater relative cost of inoculation under low light levels.

In mycorrhizal symbioses, the host plant tends to gain less from trading the carbon for the mycorrhiza-derived nutrients if the soil nutrients are in high supply (Koide, 1991; Schwartz and Hoeksema, 1998; Jones and Smith, 2004; Hoeksema et al., 2010). We conducted a series of experiments, again with a large numbers of senior undergraduate students, in which model plant *A. thaliana* responses to DSE inoculation were evaluated under different environmental conditions. These studies indicate that the host responses to inoculation are insensitive to the nutrient availability (50% greater addition of Murashige and Skoog basal salt mixture) or elevated temperatures (∼5°C increase using a horticultural heating mat) as inferred from the non-significant interactions between the inoculation and environmental variable (data not shown). In contrast – and as predicted by the *mutualism-parasitism-continuum* paradigm – experiments in which energy flow (light) into the system was controlled by shading (half of the experimental units were individually covered with a horticultural shade cloth) indicate that the relative cost of symbiosis increases when availability of light and resultant energy is reduced (**Figure 5**). These experiments utilized a petri plate design identical to those in Mandyam et al. (2013) and illustrate the ease of conducting model plant experiments that permit testing hypotheses on environmental modulation of host responses expediently under tightly controlled experimental conditions.

## THE MODEL

**FIGURE 6:**
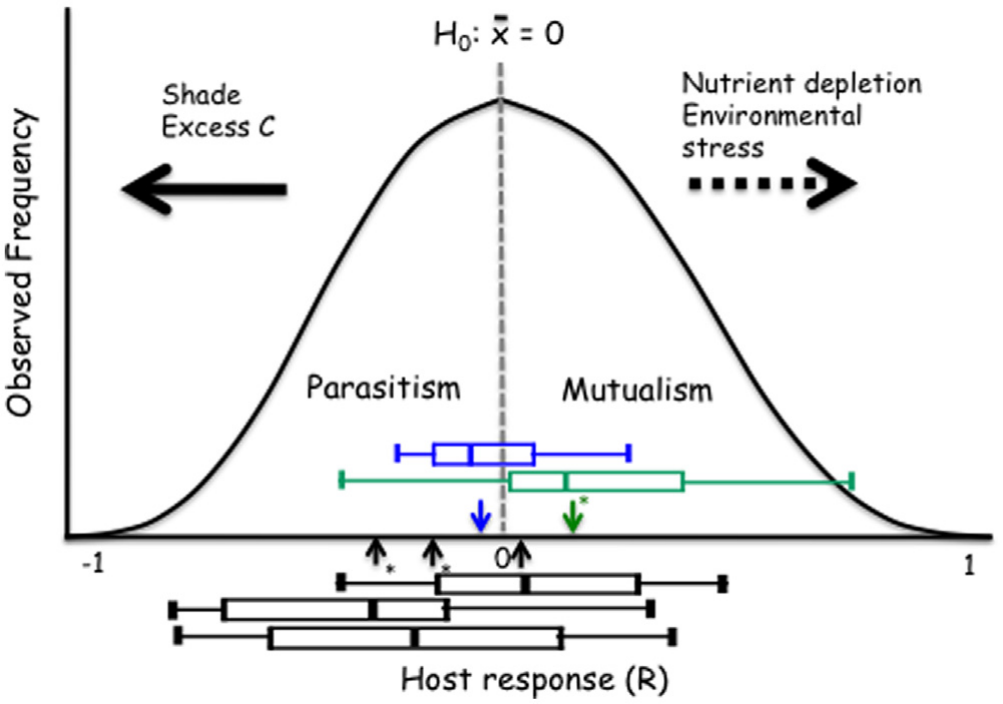
**Proposed model with the null hypothesis (H_**0**_) of no response to inoculation.** The black boxes show the range of responses of the three *Arabidopsis* accessions **(**Figure 3**)**. The green and blue boxes identify grass and forb responses, respectively. Horizontal arrows show predicted (dashed) or observed and supported (solid) responses to environmental controls. The boxes identify median, quartiles and 95% confidence intervals for three *Arabidopsis* accessions (Cvi-1, Col-0, and Kin-1 from left to right), grass (green) and forb (blue) experiments. Arrows indicate the mean response for the *Arabidopsis*, grass and forb experiments; asterisks indicate when the mean is different from zero (*t*-test; *P* < 0.05).

We propose a model that provides insight into how the host response to DSE fungi depends on the host species or ecotype and how these relationships respond to environmental variability. This model can be generally utilized for evaluation of the *mutualism-parasitism-continuum* paradigm. The proposed model rests on an assumption that – overall – the host responses to conspecific individuals drawn at random from a population of endophytic fungi are approximately normally distributed (**Figure 6**). It is of note that the larger model plant data sets generally support this assumption (**Figure 1**). This model also allows for an explicit articulation of the null-hypothesis of no response to inoculation and subsequent evaluation of this null-hypothesis. While it is not possible to predict host’s response to any one fungal strain/individual, the responses may range from strong inhibition or promotion of host performance and an average response for the population can be estimated (see **Figure 1**). Analyses of the model and native plant data strongly indicate that both positive and negative responses occur. Further, the overall, average response to a population of fungi or across host ecotypes can be evaluated by testing the general null-hypothesis that the mean host response equals zero. Average host responses that exceed zero can be considered mutualisms, whereas a negative mean response suggests a parasitic, non-beneficial association (**Figure 6**).

Our model also allows evaluation and visualization of hypotheses on environmental modulation of these symbioses. The empirical data presented above show that – consistently with the mutualism–parasitism paradigm – reduction in the light levels shift the outcome of the symbiosis toward parasitism. While there is no empirical data to support shifts toward mutualism, increasing nutrient depletion (e.g., P for AM symbiosis) or environmental stress (temperature in *Dichanthelium–Curvularia* symbiosis; Redman et al., 1999) can be predicted to lead to a greater benefit derived from symbiosis (**Figure 6**).

The empirical data and the model that we present here focus on root-associated DSE fungi. However, the model and its predictions are applicable more broadly to other host-fungus associations. The general model proposed here serves as a general tool to visualize and evaluate outcomes of symbioses when adequate numbers of conspecifics can be drawn from a population. We envision the use of this model and the predictions on the shifts as a result of environmental modulation to be particularly valuable when the outcomes of symbioses are evaluated under shifting environmental conditions. The proposed model thus allows for visualization of the variable host responses to a population of fungi and the modulation of these responses when the environmental conditions change.

## DISSECTING THE DSE SYMBIOSIS USING GENOMIC TOOLS

While the host growth responses to fungal inoculations have dominated research in the past, the next-generation chip and sequencing tools have revolutionized the depth at which the symbioses can be queried. Transcriptome analyses of various plant-microbe symbioses including many mutualisms [arbuscular mycorrhiza (AM), ectomycorrhiza (ECM), plant growth promoting bacteria (PGPR), nitrogen fixing bacteria] have been dissected by the use of microarrays available for model plants (e.g., *Arabidopsis*, tomato, maize, wheat, *Medicago*, soybean). Examples of such studies include AM-tomato (Fiorilli et al., 2009; Salvioli et al., 2012); AM-*Medicago* (Hohnjec et al., 2005; Küster et al., 2007); legume root nodulation – soybean-*Bradyrhizobium japonicum* (Brechenmacher et al., 2008) or *Medicago* nodulation (El Yahyaoui et al., 2004; Küster et al., 2004); ECM symbiosis (Johansson et al., 2004; Le Quéré et al., 2005); *Frankia*–*Alnus* symbiosis (Alloisio et al., 2010); *Arabidopsis*–*Trichoderma sp.* (Mathys et al., 2012; Morán-Diez et al., 2012; Brotman et al., 2013); *Arabidopsis*-PGPR (Wang et al., 2005; Lakshmanan et al., 2013; Spaepen et al., 2014); fungal or viral pathogens of *Arabidopsis* (Postnikova and Nemchinov, 2012; Pierce and Rey, 2013; Schuller et al., 2014); and, wheat-powdery mildew (Xin et al., 2012) or wheat-*Fusarium* head blight (Golkari et al., 2007).

Despite the availability of innumerable molecular tools for *Arabidopsis*, its use to query root-associated, mycorrhizal symbioses is difficult because *Arabidopsis* is inherently non-mycorrhizal. However, the recent discovery of non-mycorrhizal Sebacinalean fungal symbiosis in *Arabidopsis* (Weiss et al., 2011), the *Arabidopsis* mutualism with *Piriformospora indica* (Peskan-Berghöfer et al., 2004), and the susceptibility of *Arabidopsis* to colonization of variety of endophytes (Garcia et al., 2013; Mandyam et al., 2013) have facilitated the use of the *Arabidopsis* model for studying such fungal symbioses. It must be kept in mind that host colonization occurs despite a sophisticated plant immune system, likely suggesting a defined discrimination against potential pathogens and simultaneous facilitation mutualist and commensal colonization (Lundberg et al., 2012).

The *Arabidopsis*–*Piriformospora* model has permitted the characterization of unique biphasic colonization mechanism of *Piriformospora* hitherto unknown in other symbioses, extensive role of plant hormones in defense signaling, induced systemic resistance, mechanisms of growth promotion, and differential gene expression during colonization (see review in Mandyam and Jumpponen, 2014). This model has provided vital insights into mechanisms that maintain this mutualism: *P*. *indica* colonization (i) induces production of indole-3-acetaldoxime (IAOx)-derived compounds in the early stages of colonization (Nongbri et al., 2012) and elevates cellular Ca^2+^ for production of IAOx-derived metabolites (Vadassery et al., 2009a); (ii) suppresses defenses involved in oxidative burst by invoking the ‘PLD-PDK1-OXI1’ (phospholipase D, 3-phosphoinosilide-dependent kinase, oxidative signal inducible 1) cascade by triggering phosphatidic acid synthesis and upregulating OXI1 and PDK genes (Camehl et al., 2011); (iii) upregulates genes MDAR2 (monodehydroascorbate reductase) and DHAR5 (dehydroascorbate reductase) of the ascorbate–glutathione cycle offering protection from oxidative burst and suppressing defense gene expression that can shift the interaction from mutualism to parasitism (Vadassery et al., 2009b); and, (iv) controls ethylene signaling (Camehl et al., 2010; Khatabi et al., 2012).

Host metabolism and nutrition can also control the fungal interaction with the host. The control on fungal lifestyle and colonization strategies is exemplified by the generalist *P*. *indica*’s symbiosis with *Arabidopsis* and *Hordeum vulgare* (barley; Lahrmann et al., 2013). Although the symbiosis is generally beneficial, *P*. *indica* maintains a predominantly biotrophic lifestyle in *Arabidopsis*. Contrastingly, in *Hordeum*, *P*. *indica* switches from biotrophy during early colonization phase (3 days post-inoculation – dpi) to saprotrophy during late colonization phase (14 dpi). The host-dependent fungal lifestyles or colonization strategies adopted by *P*. *indica* in respective hosts are accompanied by (i) cytological distinctions including the formation of secondary thin hyphae (SH), host cell wall appositions (papillae) and host cell death and autofluorescence in *Hordeum* – whereas in *Arabidopsis*, SH, papillae and host cell death are absent; (ii) transcriptional changes in *P*. *indica* with (a) larger number of fungal genes differentially regulated during early colonization in *Arabidopsis* than in *Hordeum* and vice-versa during late colonization; (b) induction of larger number of fungal effectors such as small secreted proteins (SSPs that control colonization by targeting host defense signal transduction and metabolism) in *Hordeum* than in *Arabidopsis*, most of which encoded for hydrolytic enzymes in *Hordeum* especially during late colonization phase; (c) lesser expression of fungal genes involved in host cell wall and lipid degradation in *Arabidopsis* than in *Hordeum*; (d) induction of fungal amino acid biosynthesis genes in *Arabidopsis* and cell wall polysaccharide metabolic genes in *Hordeum*, coinciding with late colonization phase; and, (iii) distinctly different fungal nitrogen metabolism in *Hordeum* and *Arabidopsis* during late colonization phase exemplified by (a) differences in fungal PiAMT1 (a high-affinity ammonium transporter and downstream signaling under N starvation) expression and (b) free amino acid levels in the host: in *Arabidopsis*, low fungal PiAMT1 expression and high free host amino acid concentrations suggest *Arabidopsis* supply of nitrogen – especially asparagine and glutamine – to the fungus, whereas in *Hordeum*, high PiAMT1 expression and low free host amino acid concentrations – especially asparagine and glutamine – indicated onset of N starvation to the fungus and coincided with the switch to saprotrophic lifestyle. RNAi silencing studies further support the conclusions of host metabolism control on fungal lifestyles during the intracellular colonization. Inhibition of fungal PiAMT1 expression by RNAi did not alter the biotrophic fungal colonization in *Arabidopsis* symbiosis implying host nitrogen supply to fungal symbiont. In contrast, RNAi suppressed-PiAMT1 *P*. *indica* symbiosis with *Hordeum* during the late colonization was accompanied by increased fungal colonization and increased free host amino acids resulting in a prolonged the fungal biotrophic phase. Overall, studies by Lahrmann et al. (2013) show that fungal nitrogen sensor (PiAMT1 specifically) is not required for biotrophic growth but mandatory for the switch from biotrophy to saprotrophy. These results imply that fungal recognition of host metabolic cues to modulate lifestyle strategies in a dynamic environment. As summarized in Mandyam and Jumpponen (2014), the detailed molecular dissection of *P. indica* mutualism was largely attributable to the simplicity of growing the hosts and availability of a variety of molecular tools, mutants, and databases for *Arabidopsis*.

The DSE symbiosis and its range within the *mutualism-parasitism-continuum* remain unresolved; the *Arabidopsis* model likely serves among the optimal candidates to shed further light toward better resolving this symbiosis. *Arabidopsis* hosts a large number of bacteria and fungi (Lundberg et al., 2012; Bodenhausen et al., 2013; Mandyam and Jumpponen, 2014) and forms DSE symbiosis in the laboratory, greenhouse, and field (Mandyam et al., 2013). Preliminary analyses of the differential gene regulation of the *Arabidopsis*-DSE symbiosis using Affymetrix ATH1 microarrays suggested that this interaction is perhaps most similar to *Trichoderma* symbiosis and/or root endophytes including rhizobacteria and mycorrhizae (Mandyam and Jumpponen, 2014). These symbioses appear to share considerable similarities in the types of upregulated genes and include many involved in metabolism, hormonal control, stress, and defenses. However, further in-depth studies similar to those conducted with *P*. *indica* are required to further dissect the DSE symbiosis. The studies conducted with *Piriformospora* and model plants are likely applicable and serve as a model to design informative new experiments to address specific aspects of other endophyte symbioses. For example, the colonization mechanism and biotrophic lifestyle of a rice DSE fungus *Harpophora oryzae* was concluded to be similar to that of *P*. *indica* (Lahrmann et al., 2013; Su et al., 2013; Xu et al., 2014).

The introduction of next generation sequencing (NGS) technologies has opened a great potential to expediently and cost-effectively explore genomics and transcriptomics of non-model plants and/or fungi. To exemplify, Sebastiana et al. (2014) used 454-pyrosequencing to analyze the transcriptome of cork oak, *Quercus suber*, in symbiosis with the ectomycorrhizal fungus *Pisolithus tinctorius.* They observed more than 2,000 genes that were differentially regulated in mycorrhizal roots compared to non-mycorrhizal controls. The fungal colonization altered root cell wall biosynthesis (short root formation and lateral root hair decay), altered flavonoid biosynthesis, and activated secretory pathways. Importantly, the expression of many genes with putative roles in nutrient transfer were altered (upregulation of genes involved in hexose transport and delivery to apoplast plus genes involved in starch biosynthesis and metabolism; activation of genes involved in nitrogen assimilation; upregulation of sugar transporters; downregulation of ammonium, most amino acid transporters, and inorganic phosphate transporters; and, upregulation of a polyamine transporter). Additionally, several plant defense genes were differentially regulated and represented categories similar to those in other symbioses such as AM and nitrogen fixing root nodules. Recently, additional studies utilizing NGS technologies have revealed the likely evolution of mutualistic DSE fungus (*H. oryzae*) from a pathogenic ancestor: Xu et al. (2014) found (i) genome of *H*. *oryzae*, a mutualistic DSE of rice (Yuan et al., 2010; Su et al., 2013), to be 8% larger than closely related plant pathogens (*Magnaporthe oryzae*, *Magnaporthe poae,* and *Gaeumannomyces graminis*); (ii) high degree of macrosynteny between *H*. *oryzae* and *M*. *poae* or *G*. *graminis* with ancestral state reconstruction analyses suggesting that divergence of hosts resulted in differentiation among the pathogens (*M*. *oryzae*, *M*. *poae*, *G*. *graminis*) and the endophyte (*H*. *oryzae*); (iii) high number of transposable elements in *H*. *oryzae* likely driving *H*. *oryzae* genome evolution; (iv) loss of 73% of genes in ‘lipid transport and metabolism’ cluster likely required for appressorium-mediated colonization of leaves in the endophyte *H*. *oryzae* compared to the pathogen *M*. *oryzae*; (v) differences in the number of G-protein-coupled receptors suggesting differing responses of *H*. *oryzae* and *M*. *oryzae* to host extracellular signals; (vi) differences in nutritional preferences of *H*. *oryzae* and *M*. *oryzae* with opposite expression patterns of cell wall-degrading enzymes; (vii) differential expression of defense related-genes in *H*. *oryzae* and *M*. *oryzae* with suppression of virulence-related genes in *H*. *oryzae*; and, (viii) the ability of *H*. *oryzae* to trigger plant hormone production and the resultant growth promotion. Studies such as these demonstrate the great promise that the rapidly evolving genome and transcriptome analysis tools bear for detailed dissection of the endophyte symbioses.

The efforts that combine model plants and genomic tools are likely to further our understanding of DSE symbiosis and clarify the DSE interaction with host plants with regard to the *mutualism-parasitism-continuum*. Unlike AM, DSE fungi are not phylogenetically cohesive. Thus, genomic studies with only a handful of taxa can obfuscate their lifestyle designation. It is important to bear in mind the diversity and complexity of the associations included into the DSE symbioses. For example, *P. fortinii* s.l-*Acephala applanata* complex (PAC) is the dominant group of DSE fungi in conifers of Northern Hemisphere (Stoyke et al., 1992; Addy et al., 2000). In contrast, *P*. *macrospinosa* and its close relatives are likely the most common DSE fungi in North American and European grasslands (Mandyam et al., 2010; Knapp et al., 2012). Including a variety of taxa and individuals from target ecosystems is the key to drawing meaningful inferences on a broad and likely diverse guild of fungi. Collections of large numbers of conspecific DSE fungi from an ecosystem are valuable in clarifying these obscure endophyte symbioses. For example, transcriptome characterization and comparisons of conspecific DSE fungi eliciting distinct growth responses on *Arabidopsis* ecotypes (Mandyam et al., 2013) can provide molecular clues about the relative importance of the host and fungal genotype controls over the outcome of symbiosis. Genomic and molecular data highlight the host-dependent nitrogen metabolism in the control of fungal lifestyle (Lahrmann et al., 2013). Meta-analyses have suggested that nitrogen supply likely impacts the outcome of DSE symbiosis (Newsham, 2011) and observational studies suggest that nitrogen fertilization can affect DSE colonization in the field (Mandyam and Jumpponen, 2008). These studies suggest complex and perhaps non-additive controls of DSE symbiosis: the outcomes are likely controlled in part by host genotype, in part by fungal genotype (Mandyam et al., 2013), and in part by environmental modulators. Yet, these complex systems suggest that the combined genotypic controls may prove valuable in dissecting the genomic factors involved in DSE nitrogen metabolism. Finally, the wealth of naturally occurring pairings of DSE fungi and host plants can provide insightful and beneficial experimental tools to ground-truth the conclusions from model systems. The exponential advances in NGS technologies permit the expedient and cost-effective genomic interrogations of the DSE symbiosis in model and non-model plants alike.

## CONCLUSION

Here we present arguments based on host growth responses and the potential for molecular dissection of an obscure endophyte symbiosis to better elucidate the ecological and molecular drivers underlying host responses to poorly known fungal symbionts. Our extensive experiments with model and non-model plants indicate a distribution of host responses to colonization and led to a proposal of a null model that permits testing hypotheses on host responses to a population of endophytic fungi as well as generating easily testable hypotheses on the shifts in these responses under altered environmental conditions. We further highlight examples of recent studies that have identified molecular cues and mechanisms underlying the host responses to fungal symbionts and vice-versa. It is the combination of the power of simple model systems and the ground-truthing those conclusions in relevant native plant systems that are likely to best elucidate the drivers and mechanisms of obscure and poorly understood symbioses. The findings of these studies can be coupled with deep interrogations of host and fungal transcriptomes to elucidate the mechanisms that underlay the observed host growth responses.

## Conflict of Interest Statement

The authors declare that the research was conducted in the absence of any commercial or financial relationships that could be construed as a potential conflict of interest.
